# Supplementary material for: Skin lesion classification using multi-resolution empirical mode decomposition and local binary pattern
Source: PLoS One. 2022 Sep 20;17(9):e0274896. doi: 10.1371/journal.pone.0274896 (PMC9488768; doi:10.1371/journal.pone.0274896)
Supplement: Attachment — Submitted filename: Response to Reviewers.docx [file pone.0274896.s001.docx]

**Response to reviewer**

Reviewer #1: Comments:

1. The used dataset is very small, it contains only 70 images of each class for training. Would that be enough to justify the results?

- The data is sufficient since we are using conventional classifiers rather than CNN with deep learning. Therefore, we believe the results are significant.

2. It is understandable that the authors used a reduced dataset from HAM10000 data for training to balance the classes. However, the testing could have been done on all the available data. Why have they chosen only 315 images for testing?

- There are many researchers who choose to separate the training and test images. In fact, we hope that separating the training and test images would allow us to see whether the ANN manages to learn the underlying pattern of each class of the training data and then generalize it to the test data that it has not seen before.

3. The Novelty/contribution of the paper is not clear. The achieved accuracy is very good but what lead to the improved accuracy, as compared to other papers. Was it the feature extraction, segmentation, or classification method?

- The novelty comes from using features from the MREMD in addition to features from the traditional LBP to help improve the accuracy. On top of that we also compare the performances of a few different classifiers

4. Figure 1 is not readable. The resolution needs significant improvements.

- Corrected as suggested.

5. The Resolution of all figures needs improvement.

- Improved as suggested

6. The literature review is weak, Authors should include more recent relevant publications and compare their results with the results of recent publications. For example, these papers should be included

i. Saeed, J. and Zeebaree, S., 2021. Skin lesion classification based on deep convolutional neural network architectures. Journal of Applied Science and Technology Trends, 2(01), pp.41-51.
ii. Javaid, Arslan, et al. "Skin Cancer Classification Using Image Processing and Machine Learning." 2021 International Bhurban Conference on Applied Sciences and Technologies (IBCAST). IEEE, 2021.
iii. Khan, M.A., Muhammad, K., Sharif, M., Akram, T. and de Albuquerque, V.H.C., 2021. Multi-class skin lesion detection and classification via teledermatology. IEEE journal of biomedical and health informatics, 25(12), pp.4267-4275.
iv. Anand, V., Gupta, S., Koundal, D., Nayak, S.R., Nayak, J. and Vimal, S., 2022. Multi-class Skin Disease Classification Using Transfer Learning Model. International Journal on Artificial Intelligence Tools, 31(02), p.2250029.

- Added as recommended

Reviewer #2:

The paper provides nothing new, all proposed methods and data are found in the literature. Moreover, the extracted features are not new and they are too much for the dataset. I think authors should combine these methods with deep learning methods rather than conventional machine learning only.

- Since the data are limited, it is not suitable to use deep learning for classification since it requires a lot of data to properly train. Furthermore, the classification rates achieved by simple conventional classifiers are high that resorting to deep learning is unnecessary.
